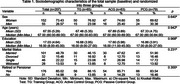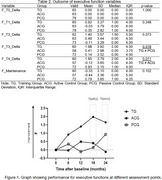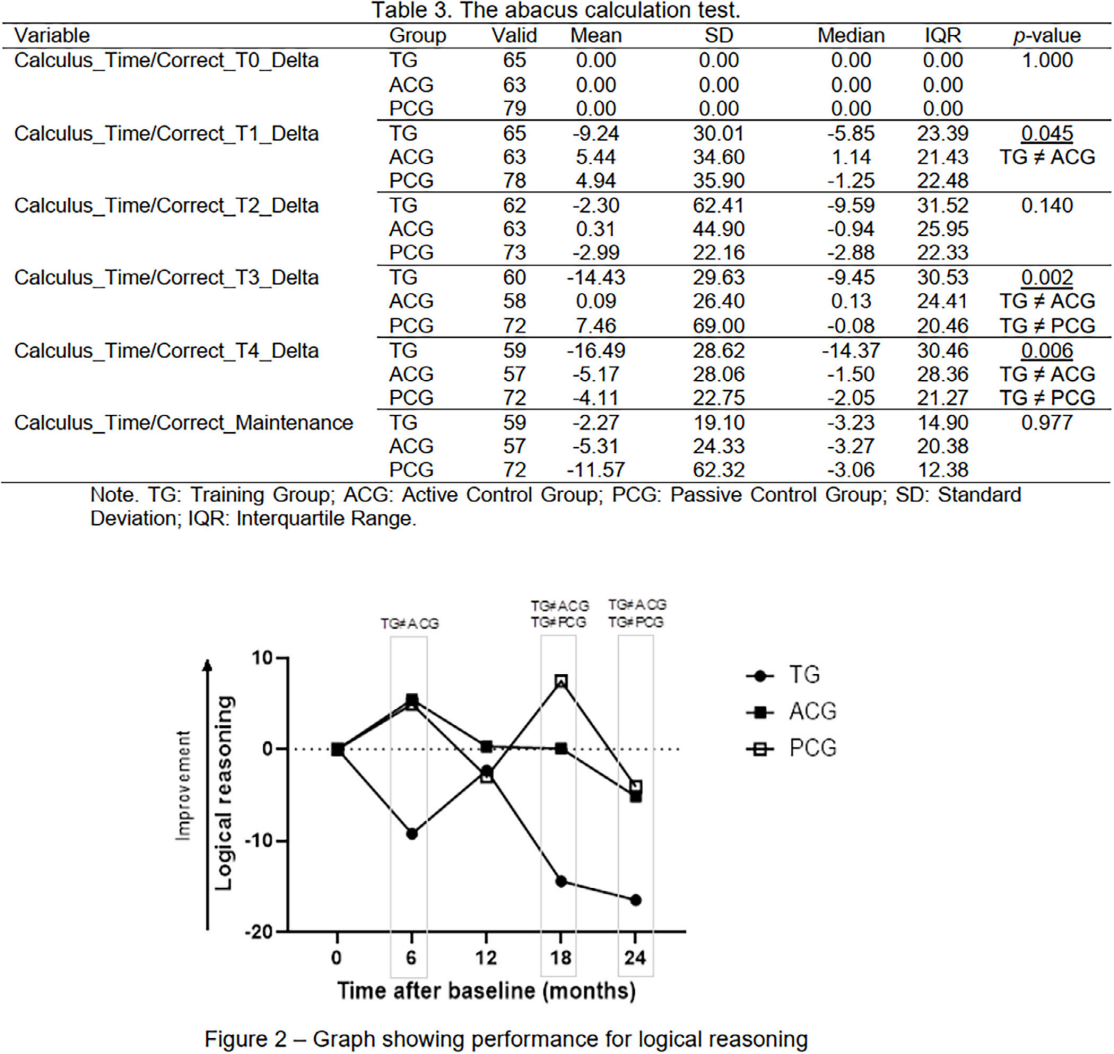# Supera Cognitive Stimulation Study effectiveness of a multicomponent cognitive intervention for cognitively unimpaired older adults: a randomized clinical controlled trial partial data

**DOI:** 10.1002/alz70858_102134

**Published:** 2025-12-25

**Authors:** Thais Bento Lima‐Silva, Tiago Nascimento Ordonez, Gabriela dos Santos, Sabrina Aparecida Da Silva, Laydiane Alves Costa, Ana Paula Bagli Moreira, Maria Antônia Antunes Fernandes, Diana Dos Santos Bacelar, Sonia Brucki, Mônica Sanches Yassuda

**Affiliations:** ^1^ Universidade de São Paulo, São Paulo, São Paulo, Brazil; ^2^ Medical School of University of São Paulo, São Paulo, São Paulo, Brazil; ^3^ Gerontology of the School of Arts, Science and Humanities of the University of São Paulo, São Paulo, São Paulo, Brazil; ^4^ Universidade de São Paulo ‐ USP, São Paulo, Brazil; ^5^ University of São Paulo Medical School, São Paulo, Brazil; ^6^ USP, São Paulo, São Paulo, Brazil

## Abstract

**Background:**

Population aging poses challenges in terms of cognitive health, requiring effective interventions. Cognitive stimulation has shown promising results in healthy older adults. This study investigated the effects of a structured cognitive stimulation program on older adults without cognitive impairment.

**Method:**

189 older adults randomized into three groups: Training Group (TG); Active Control Group (ACG); and Passive Control Group (PCG). The primary outcome of the study was cognitive performance assessed by the following tests: Addenbrooke's Cognitive Examination‐Revised (ACE‐R); Short Cognitive Performance Test (SKT); Forward and Backward Digit Span Test; Trail Making Test A and B; Phonemic Verbal Fluency Test for the letters FAS; Abacus Calculation Test. Secondary outcomes were psychosocial variables, which included the Geriatric Depression Scale (GDS); the Depression Anxiety and Stress Scale (DASS); the Control, Autonomy, Self‐Realization and Pleasure (CASP‐19); and the Minimum Map of Relationships of Older Individuals (MRRI). The intervention consisted of cognitive stimulation 72 sessions conducted weekly for an 18‐month period, with assessments performed over 24 months.

**Result:**

Traditional analyses showed significant statistical differences on delta analyses on the verbal fluency variables letter F and calculus test, indicating improvement in the TG during the 24‐month assessment compared to the other experimental groups. Z‐scores revealed improvements in cognitive functions, such as working memory and logical reasoning. Benefits were maintained after 12 and 18 months. Also, the TG showed improvements over 24 months in the form of reduced depressive symptoms and better self‐perceived memory performance compared to the ACG and PCG. Cognitive stimulation demonstrated potential cognitive benefits, with positive implications for healthy aging. Continuous multimodal strategies may enhance long‐term effects, making this intervention a promising tool for cognitive preservation in older individuals.

**Conclusion:**

The profile of older adults assessed may have contributed to the efficacy of the training, given that many were active, socially engaged, high‐educated individuals compared with the general Brazilian older population. The low drop‐out rate demonstrates the high level of adherence to the intervention. The cognitive training was likely more attractive to participants because it sought to improve both cognitive performance and psychosocial variables concomitantly.